# Erythema Dyschromicum Perstans in an 8-Year-Old Indian Child

**DOI:** 10.1155/2018/2143089

**Published:** 2018-07-15

**Authors:** Alexander K. C. Leung, Joseph M. Lam

**Affiliations:** ^1^Clinical Professor of Pediatrics at The University of Calgary, Canada; ^2^Pediatric Consultant at The Alberta Children's Hospital, Calgary, Alberta, Canada T2M 0H5; ^3^Clinical Associate Professor of Pediatrics at the University of British Columbia, Canada; ^4^Associate Member at the Department of Dermatology and Skin Sciences at the University of British Columbia, Vancouver, British Columbia, Canada

## Abstract

We report an 8-year-old East Indian boy with erythema dyschromicum perstans. The condition has very rarely been reported in prepubertal Indian children. A perusal of the literature revealed but two cases, to which we add another one. Recognition of erythema dyschromicum perstans in prepubertal Indian children is important for proper diagnosis and to prevent unnecessary investigations.

## 1. Introduction

Erythema dyschromicum perstans, also known as ashy dermatosis or dermatosis cenicienta, is an acquired, chronic pigmentary disorder characterized by slowly progressive, ashy gray-colored macules/patches distributed symmetrically on the trunk and proximal extremities [1]. The condition was first described in 1957 by Oswaldo Ramirez in Salvadorans [2]. Ramirez called patients with this condition* los cenicienta* which in Spanish means the ash-colored ones because of the characteristic ashy color of the lesions [2]. The term “erythema dyschromicum perstans" was coined by Convit et al. in 1961 when they reported five patients with numerous macules of grayish color with slightly raised, firm erythematous border [3]. The disorder has very rarely been reported in prepubertal Indian children. A perusal of the literature revealed but two cases, to which we add another.

## 2. Case Report

An 8-year-old East Indian boy with Fitzpatrick skin type IV phototype complexion presented with numerous blue-gray macules and patches over the back, anterior trunk, arms, and legs of 8 months' duration. The lesions first appeared on the back and then spread to the anterior trunk, arms, and legs. Some of the lesions were mildly pruritic and some with preceding erythematous borders. The lesions were progressive and increased in size and number with time. There were no identifiable triggers. His past medical history was significant for Berry syndrome (a complex aortopulmonary malformation). The aortopulmonary malformation was repaired surgically at 10 days of life. The surgical repair was successful and the postoperative course was uneventful. Otherwise, his health was unremarkable and he was not on any medications. There was no history of previous skin eruption. He had no known family history of autoimmune disorder or similar skin disease.

**Figure 1:**
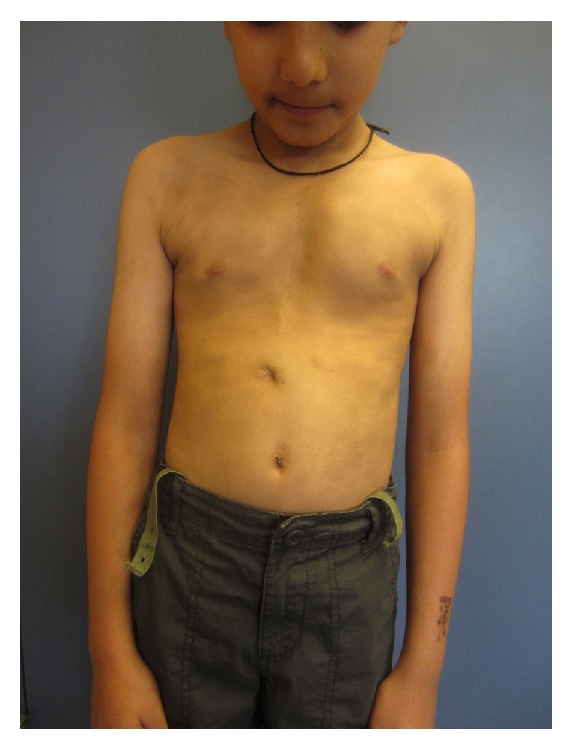
Well-demarcated oval ash-brown macules and patches symmetrically distributed over the anterior chest and abdomen.

**Figure 2:**
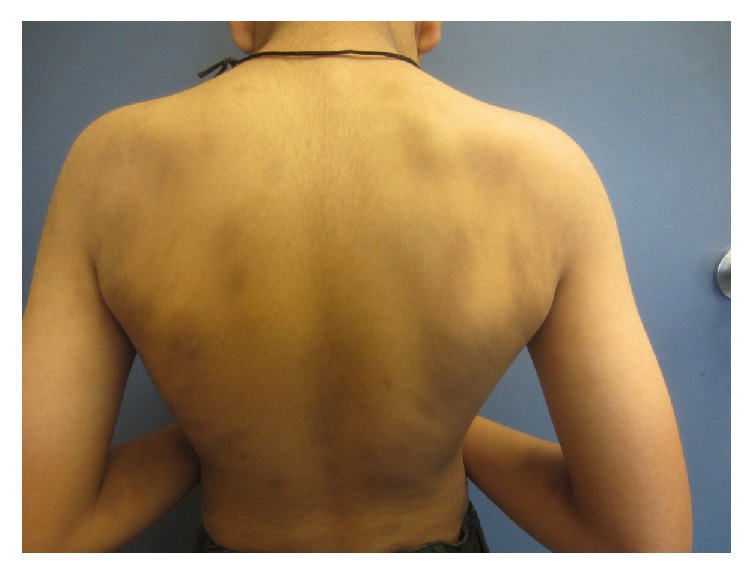
Well-demarcated oval ash-brown macules and patches symmetrically distributed over the upper back.

**Figure 3:**
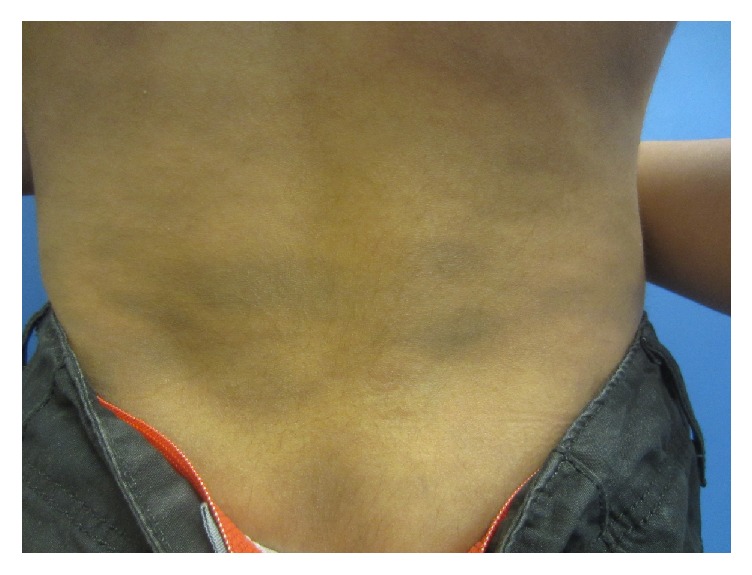
Well-demarcated oval blue-gray macules and patches symmetrically distributed over the lower back.

On physical examination, there were numerous well-demarcated, oval, ash-brown macules and patches symmetrically distributed over the back, anterior trunk, arms, and legs (Figures 1–3). The lesions measured 0.5 to 6 cm and some lesions were confluent. There were no erythematous borders and no desquamation. Darier's sign was negative. The mucous membranes, face, scalp, palms, soles, and nails were spared. A well-healed scar from previous sternotomy was noted on the chest. The rest of the physical examination was unremarkable.

**Figure 4:**
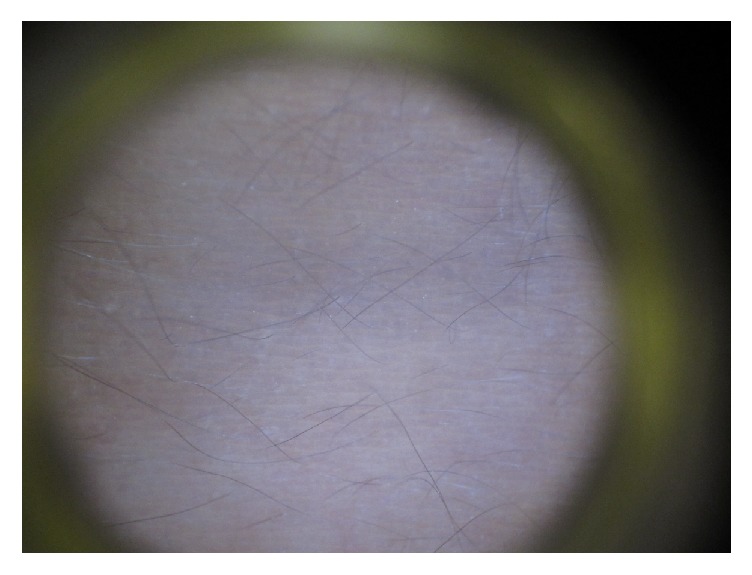
Dermoscopy of a lesion showed gray-blue to bluish small dots over a bluish background, corresponding to melanin-laden melanophages in deeper dermis (Tyndall effect).

Dermoscopy of a lesion showed faint gray-blue to bluish small dots over a bluish background, corresponding to melanin-laden melanophages in deeper dermis (Tyndall effect) (Figure 4). The patient was diagnosed to have erythema dyschromicum perstans based on the clinical and dermoscopic findings.

Parents were reassured of the benign nature of the disorder and that the lesions would resolve with time. A skin biopsy was declined by the parents.

## 3. Discussion

Typically, erythema dyschromicum perstans presents as ashy gray to grayish brown oval macules/patches symmetrically distributed over the body [4–7]. Sites of predilection include the trunk, followed by the proximal extremities, neck, and face [8]. The mucosal surfaces, genitals, scalp, palms, soles, and nails are generally spared [9]. The size of the lesions ranges from a few millimeters to several centimeters in diameter [9]. A slightly raised, erythematous border may be present in the early stage which, when present, is characteristic [10, 11]. The erythematous border tends to resolve with time [6]. In one study, an erythematous border is found only in 17.6% of cases [9]. The condition is typically asymptomatic, but mild pruritus may occur [5, 7, 9]. Dermoscopy of the lesion typically shows gray-blue to bluish small dots over a bluish background, corresponding to melanin-laden melanophages in deeper dermis (Tyndall effect), as is illustrated in the present case [12].

Our patient had typical clinical and dermoscopic features of erythema dyschromicum perstans. A skin biopsy was declined by the parents given the benign nature of the disorder and that the lesions tend to resolve with time.

Erythema dyschromicum perstans occurs most commonly in individuals under 30 years of age with a peak in the second decade of life [7, 9]. The disorder is rare in prepubertal children, especially in the Asian population [13]. In adults, the disorder is more prevalent in the Latin American and Asian population [1, 7, 9]. Dark-skinned individuals (Fitzpatrick types IV and V) are most commonly affected [1, 8, 9]. In contrast to the adult population, children with this disorder are usually Caucasians [6, 13, 14]. Erythema dyschromicum perstans has very rarely been reported in prepubertal Indian children. A perusal of the literature revealed but two cases. In 1996, Umap reported the first case of erythema dyschromicum perstans in a 10-year-old Indian child with multiple lesions of erythema dyschromicum perstans over the face and upper limbs of 10 months' duration [15]. In 2013, Keisham et al. reported another case of erythema dyschromicum perstans in an 8-year-old Indian child who presented with asymptomatic, gradually progressive ashy gray-colored, confluent, symmetrical macules over the entire trunk, proximal limbs, posterior neck, forehead, and left upper eyelid [13]. Herein, we report an 8-year-old East Indian boy with erythema dyschromicum perstans.

The exact etiology of erythema dyschromicum perstans is not known. Most cases are idiopathic [7]. Erythema dyschromicum perstans has been reported in association with exposure to drugs (penicillin, benzodiazepines, ethambutol, fluoxetine, and omeprazole), radiographic contrast media (barium sulfate), parasitic infestations (whipworm), fungicide (chlorothalonil), chemicals (ammonium nitrate), herbal consumption (Tokishakuyakusa), endocrinopathies (hypothyroidism, diabetes mellitus), dyslipidemia, infections (human immunodeficiency virus, hepatitis C, and enterovirus), and cobalt allergy [16–20]. Our patient did not have any identifiable triggers. Appropriate laboratory investigations should be performed if an underlying cause is suspected.

Erythema dyschromicum perstans should be differentiated from idiopathic eruptive macular pigmentation and lichen planus pigmentosus. In idiopathic eruptive macular pigmentation, lesions are ashy brown, nonconfluent, and smaller in size and tend to regress with time. In lichen planus pigmentosus, lesions are brown to gray-brown macules/patches, pruritic, and without an active erythematous border. The lesions often occur in sun-exposed and intertriginous areas and may involve mucous membranes. Other differential diagnoses include maculopapular mastocytosis, postinflammatory hyperpigmentation, morphea, pityriasis rosea, Addison disease, hemochromatosis, arsenism, contact dermatitis, multiple fixed drug eruption, confluent and reticulated papillomatosis of Gougerot and Carteaud, tuberculoid leprosy, and pinta.

Erythema dyschromicum perstans can be cosmetically distressing and socially embarrassing, especially if the lesion occurs in a visible area such as the face [1, 21]. The cutaneous eruption tends to resolve in 2 to 3 years in prepubertal children but tends to persist in adults [4, 6]. Our patient and family were reassured of the benign nature of the condition and that the lesions would resolve with time.

## 4. Conclusion

In adults, erythema dyschromicum perstans is more prevalent in the Latin American and Asian population. Dark-skinned individuals are most commonly affected. In contrast to the adult population, children with this disorder are usually Caucasians. Erythema dyschromicum perstans has very rarely been reported in prepubertal Indian children. A perusal of the literature revealed but two cases, to which we are going to add another one. Erythema dyschromicum perstans should be included in the differential diagnosis of pigmentary disorders in prepubertal Indian children. Recognition of erythema dyschromicum perstans in prepubertal Indian children is important so that a proper diagnosis can be made.
